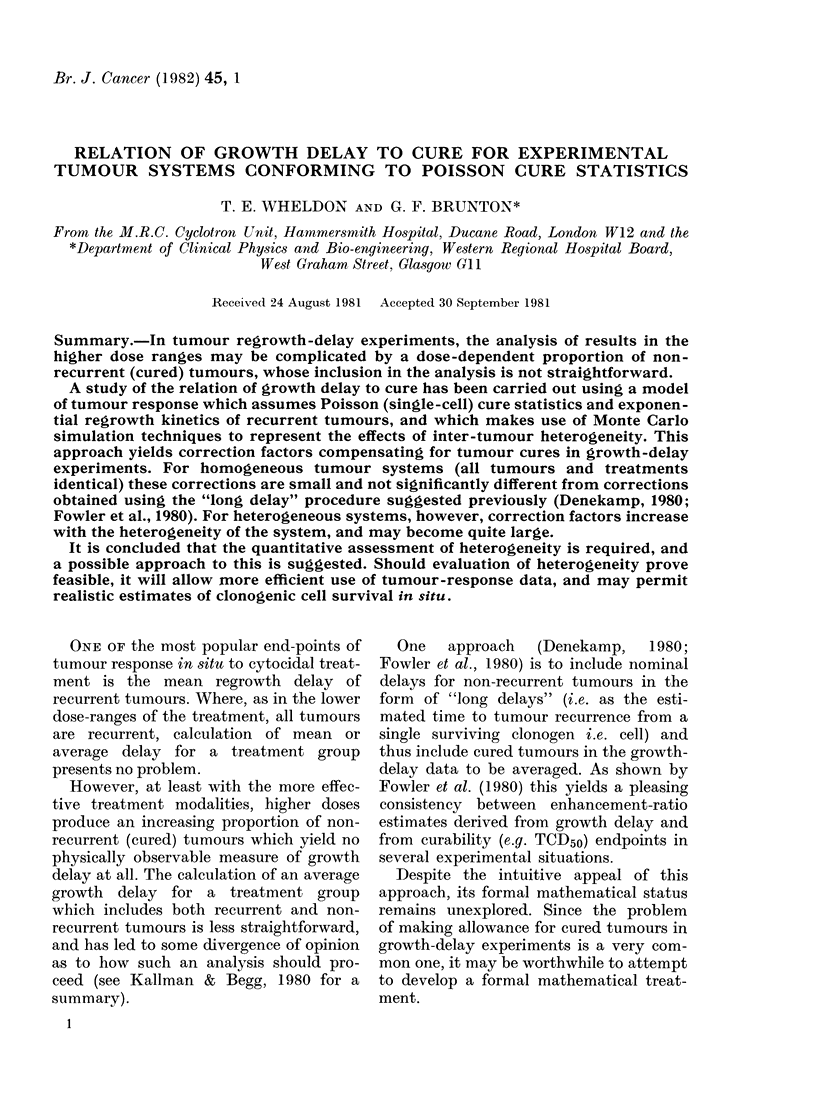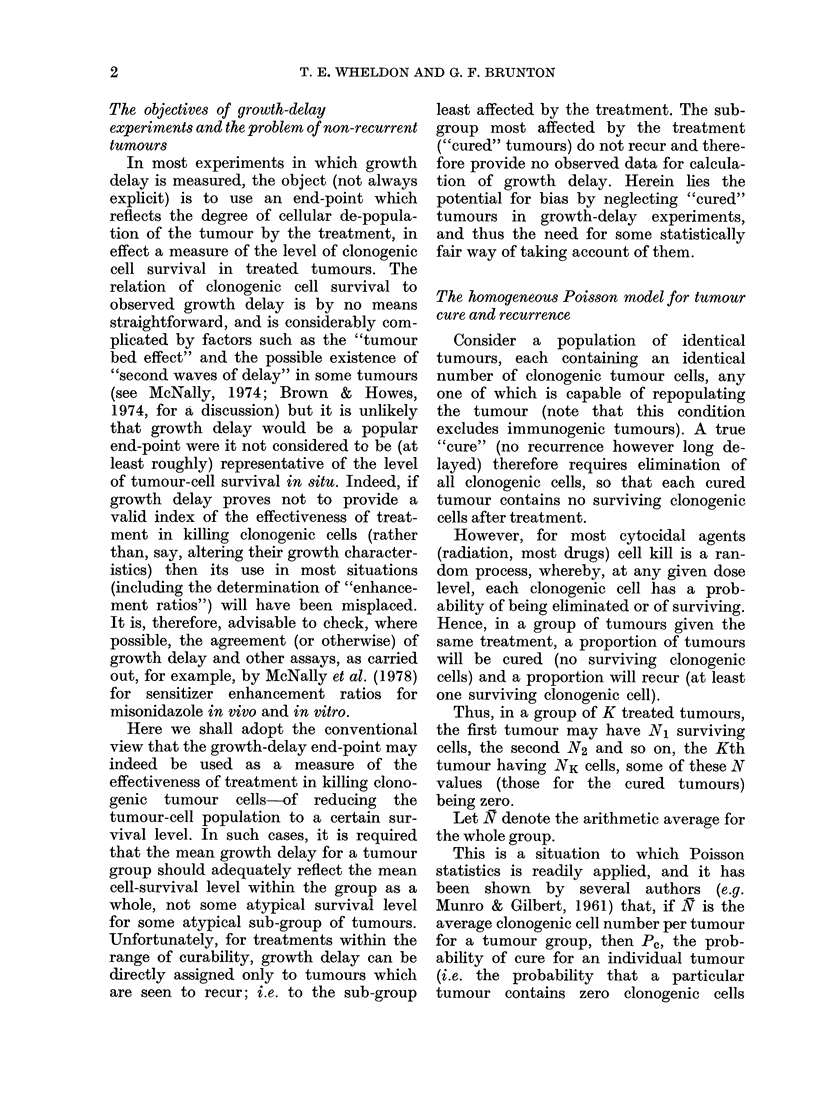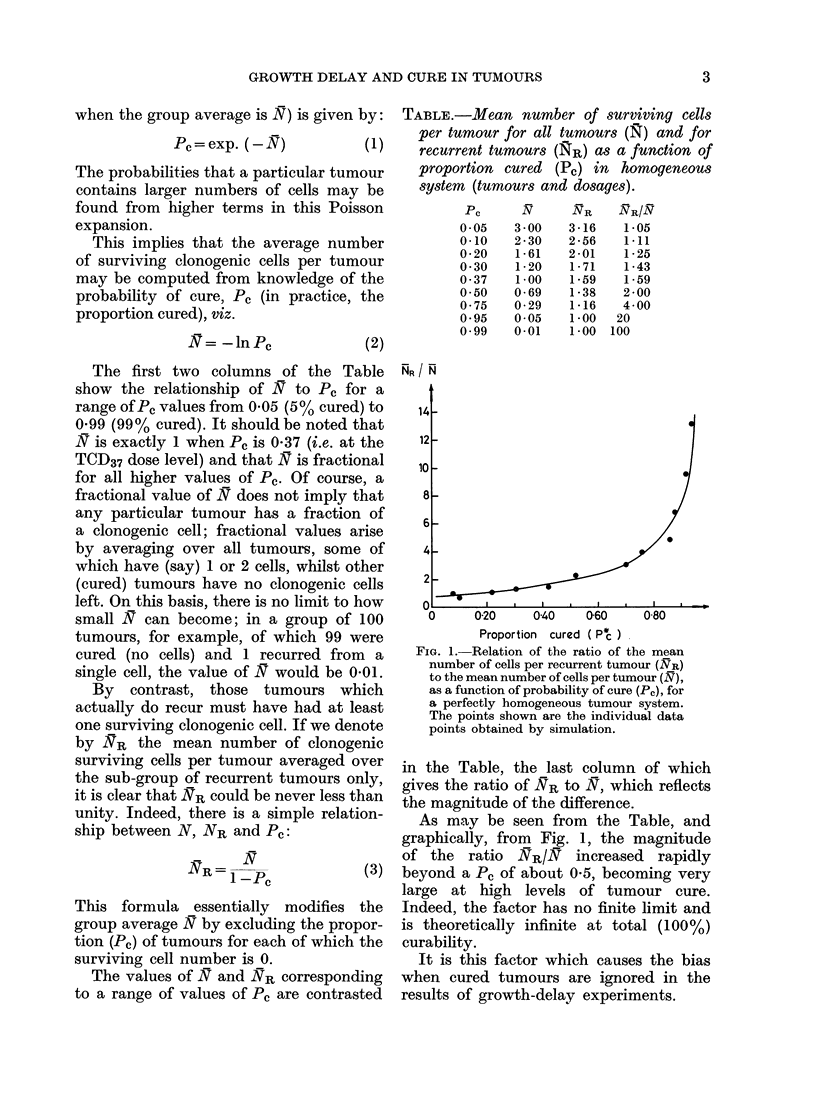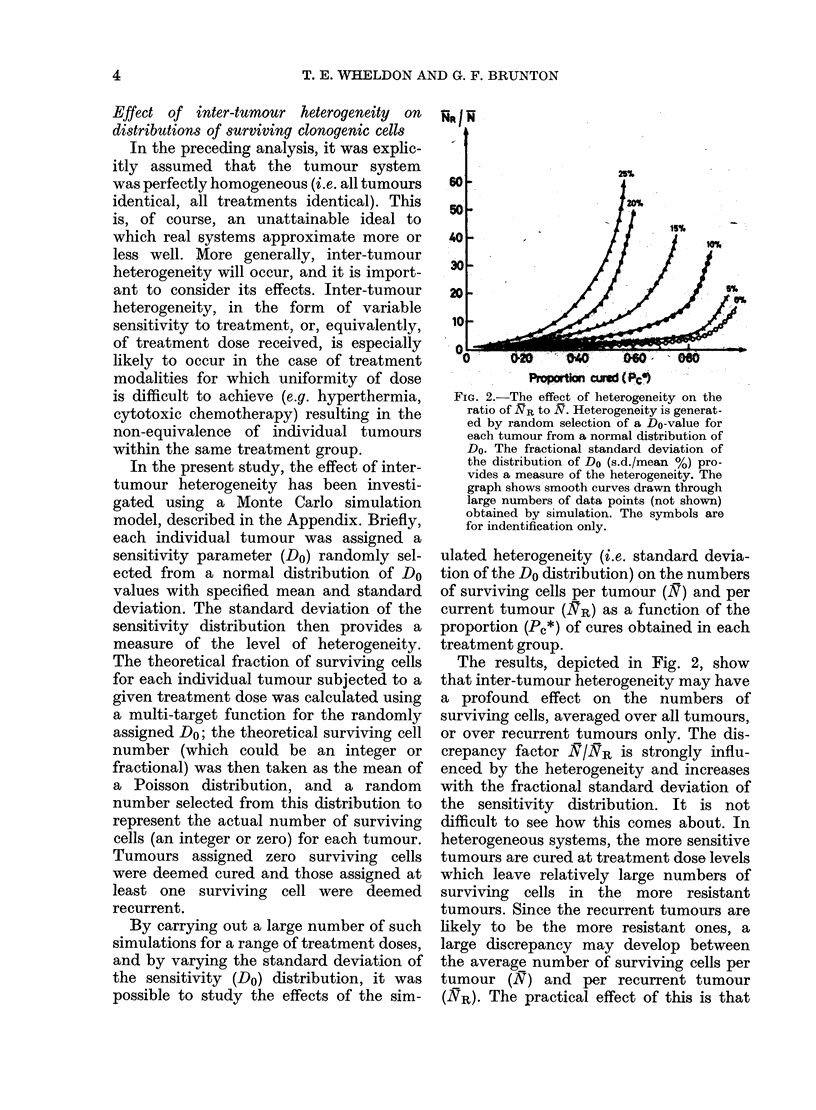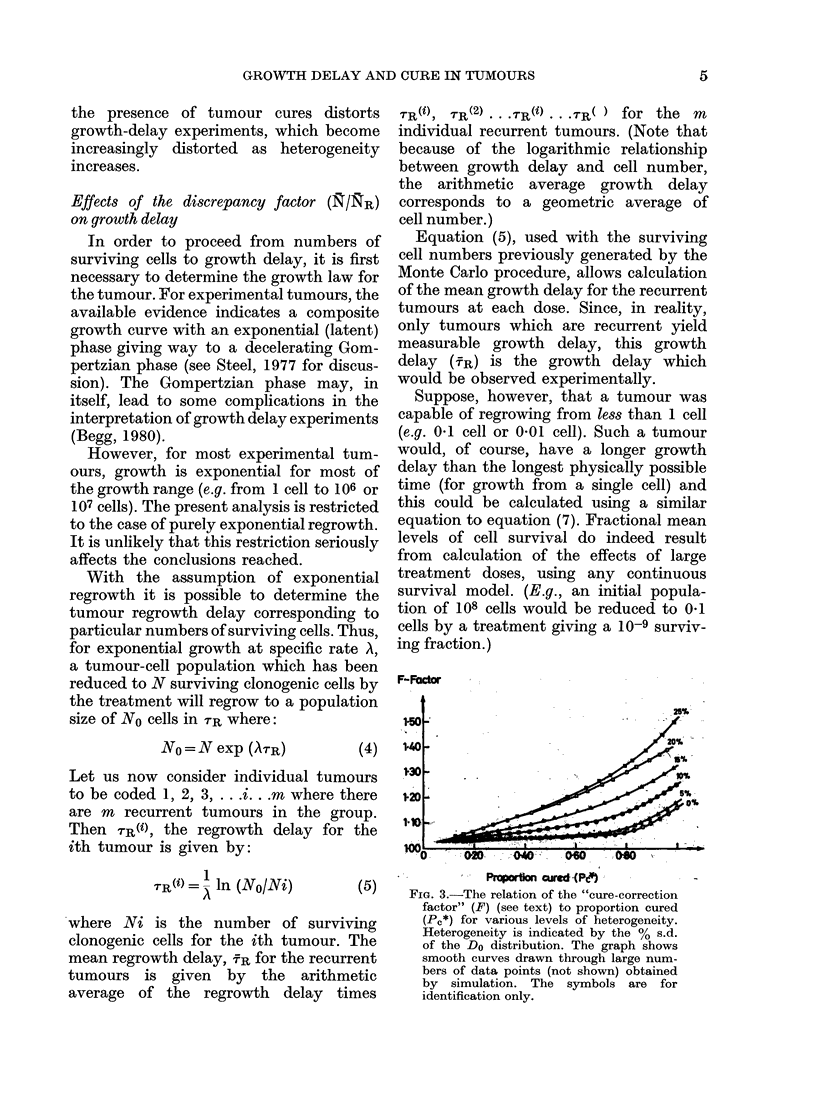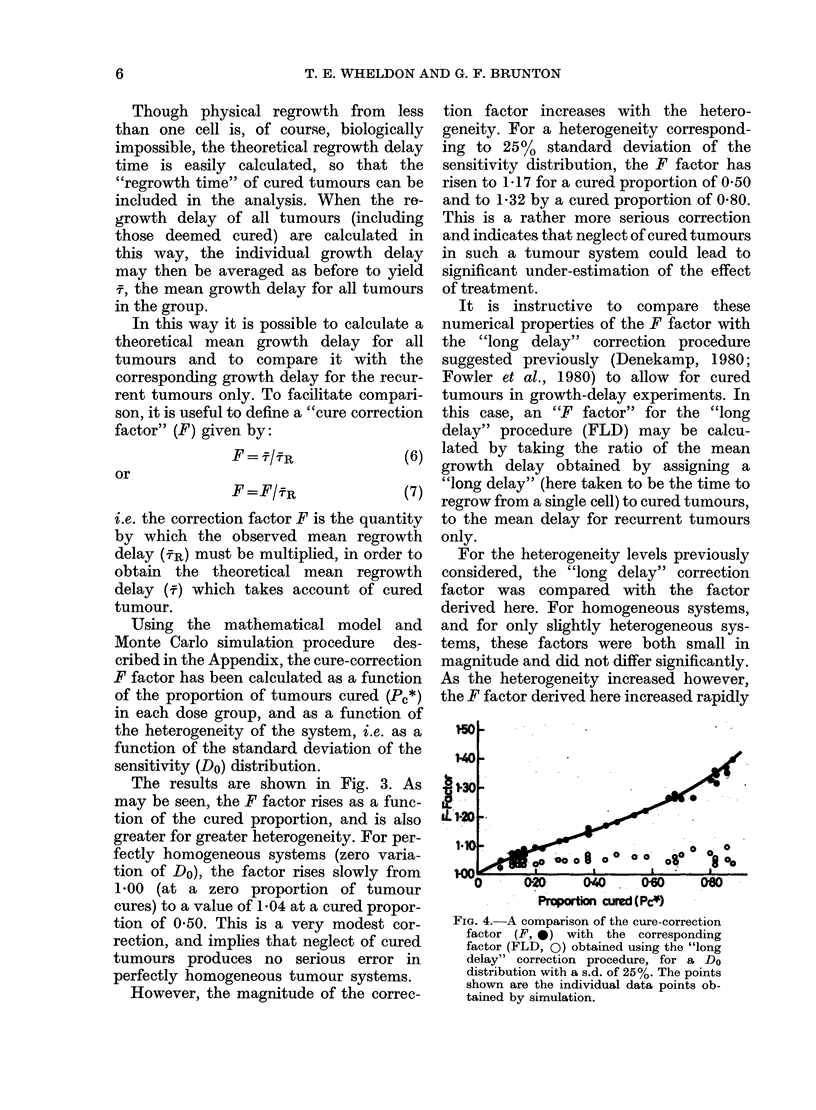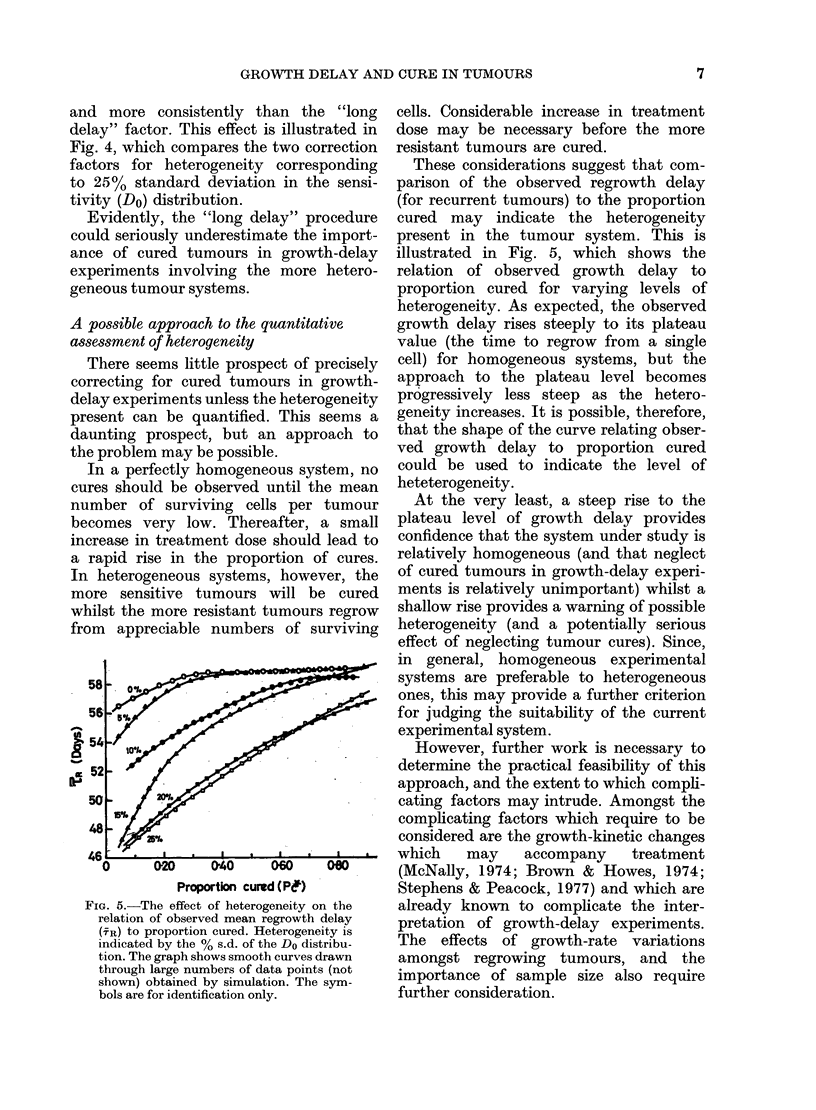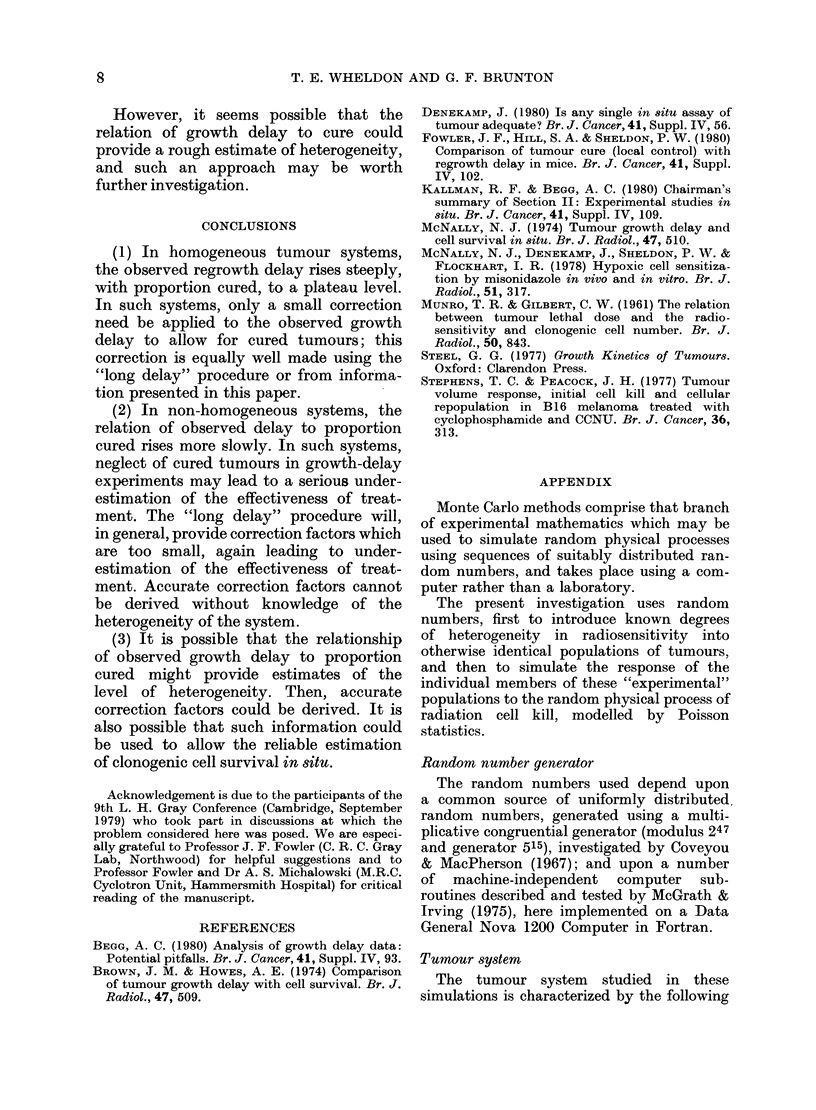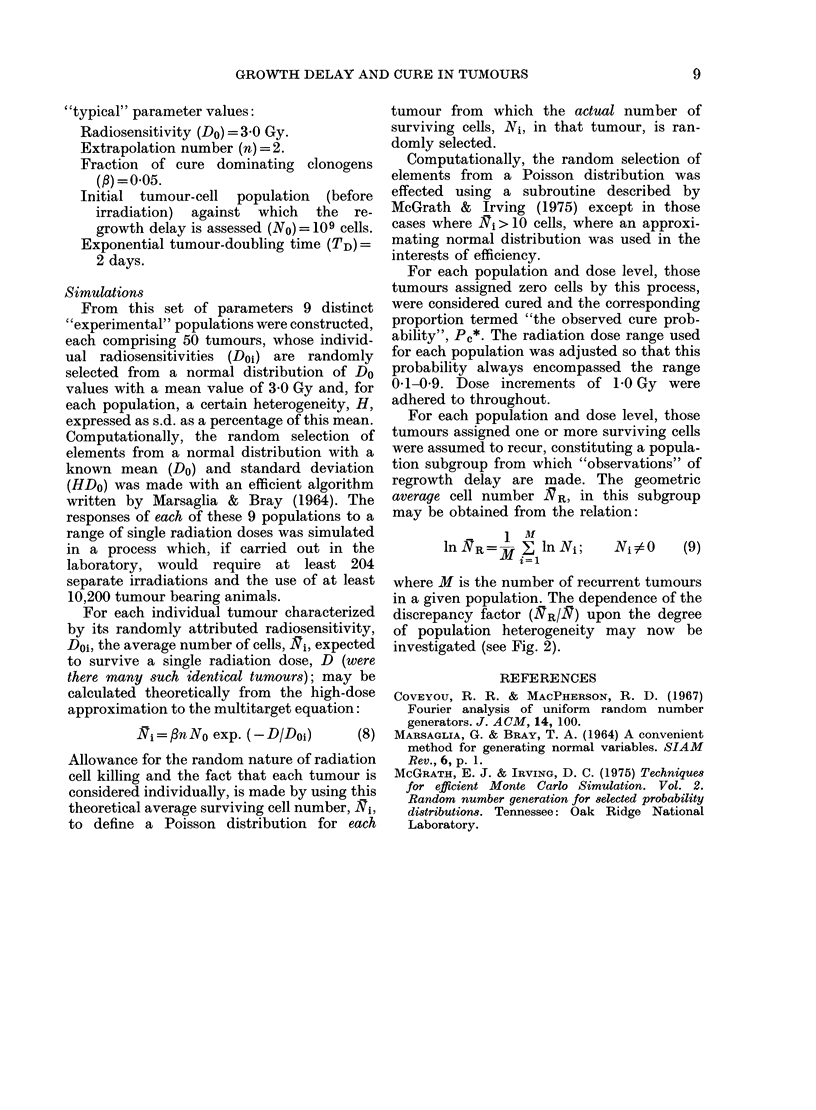# Relation of growth delay to cure for experimental tumour systems conforming to Poisson cure statistics.

**DOI:** 10.1038/bjc.1982.1

**Published:** 1982-01

**Authors:** T. E. Wheldon, G. F. Brunton

## Abstract

In tumour regrowth-delay experiments, the analysis of results in the higher dose ranges may be complicated by a dose-dependent proportion of non-recurrent (cured) tumours, whose inclusion in the analysis is not straightforward. A study of the relation of growth delay to cure has been carried out using a model of tumour response which assumes Poisson (single-cell) cure statistics and exponential regrowth kinetics of recurrent tumours, and which makes use of Monte Carlo simulation techniques to represent the effects of inter-tumour heterogeneity. This approach yields correction factors compensating for tumour cures in growth-delay experiments. For homogeneous tumour systems (all tumours and treatments identical) these corrections are small and not significantly different from corrections obtained using the "long delay" procedure suggested previously (Denekamp, 1980; Fowler et al., 1980). For heterogeneous systems, however, correction factors increase with the heterogeneity of the system, and may become quite large. It is concluded that the quantitative assessment of heterogeneity is required, and a possible approach to this is suggested. Should evaluation of heterogeneity prove feasible, it will allow more efficient use of tumour-response data, and may permit realistic estimates of clonogenic cell survival in situ.


*Br. J. Cancer* (1982) **45**, 1

### RELATION OF GROWTH DELAY TO CURE FOR EXPERIMENTAL TUMOUR SYSTEMS CONFORMING TO POISSON CURE STATISTICS

T. E. WHELDON AND G. F. BRUNTON*

*From the M.R.C. Cyclotron Unit, Hammersmith Hospital, Ducane Road, London W12 and the *Department of Clinical Physics and Bio-engineering, Western Regional Hospital Board, West Graham Street, Glasgow G11*

Received 24 August 1981 Accepted 30 September 1981

**Summary.—In tumour regrowth-delay experiments, the analysis of results in the higher dose ranges may be complicated by a dose-dependent proportion of non-recurrent (cured) tumours, whose inclusion in the analysis is not straightforward.**

**A study of the relation of growth delay to cure has been carried out using a model of tumour response which assumes Poisson (single-cell) cure statistics and exponential regrowth kinetics of recurrent tumours, and which makes use of Monte Carlo simulation techniques to represent the effects of inter-tumour heterogeneity. This approach yields correction factors compensating for tumour cures in growth-delay experiments. For homogeneous tumour systems (all tumours and treatments identical) these corrections are small and not significantly different from corrections obtained using the "long delay" procedure suggested previously (Denekamp, 1980; Fowler et al., 1980). For heterogeneous systems, however, correction factors increase with the heterogeneity of the system, and may become quite large.**

**It is concluded that the quantitative assessment of heterogeneity is required, and a possible approach to this is suggested. Should evaluation of heterogeneity prove feasible, it will allow more efficient use of tumour-response data, and may permit realistic estimates of clonogenic cell survival *in situ*.**

ONE OF the most popular end-points of tumour response *in situ* to cytocidal treatment is the mean regrowth delay of recurrent tumours. Where, as in the lower dose-ranges of the treatment, all tumours are recurrent, calculation of mean or average delay for a treatment group presents no problem.

However, at least with the more effective treatment modalities, higher doses produce an increasing proportion of non-recurrent (cured) tumours which yield no physically observable measure of growth delay at all. The calculation of an average growth delay for a treatment group which includes both recurrent and non-recurrent tumours is less straightforward, and has led to some divergence of opinion as to how such an analysis should proceed (see Kallman & Begg, 1980 for a summary).

One approach (Denekamp, 1980; Fowler *et al.*, 1980) is to include nominal delays for non-recurrent tumours in the form of "long delays" (*i.e.* as the estimated time to tumour recurrence from a single surviving clonogen *i.e.* cell) and thus include cured tumours in the growth-delay data to be averaged. As shown by Fowler *et al.* (1980) this yields a pleasing consistency between enhancement-ratio estimates derived from growth delay and from curability (*e.g.* $TCD_{50}$) endpoints in several experimental situations.

Despite the intuitive appeal of this approach, its formal mathematical status remains unexplored. Since the problem of making allowance for cured tumours in growth-delay experiments is a very common one, it may be worthwhile to attempt to develop a formal mathematical treatment.

*The objectives of growth-delay experiments and the problem of non-recurrent tumours*

In most experiments in which growth delay is measured, the object (not always explicit) is to use an end-point which reflects the degree of cellular de-population of the tumour by the treatment, in effect a measure of the level of clonogenic cell survival in treated tumours. The relation of clonogenic cell survival to observed growth delay is by no means straightforward, and is considerably complicated by factors such as the "tumour bed effect" and the possible existence of "second waves of delay" in some tumours (see McNally, 1974; Brown & Howes, 1974, for a discussion) but it is unlikely that growth delay would be a popular end-point were it not considered to be (at least roughly) representative of the level of tumour-cell survival *in situ*. Indeed, if growth delay proves not to provide a valid index of the effectiveness of treatment in killing clonogenic cells (rather than, say, altering their growth characteristics) then its use in most situations (including the determination of "enhancement ratios") will have been misplaced. It is, therefore, advisable to check, where possible, the agreement (or otherwise) of growth delay and other assays, as carried out, for example, by McNally *et al.* (1978) for sensitizer enhancement ratios for misonidazole *in vivo* and *in vitro*.

Here we shall adopt the conventional view that the growth-delay end-point may indeed be used as a measure of the effectiveness of treatment in killing clonogenic tumour cells—of reducing the tumour-cell population to a certain survival level. In such cases, it is required that the mean growth delay for a tumour group should adequately reflect the mean cell-survival level within the group as a whole, not some atypical survival level for some atypical sub-group of tumours. Unfortunately, for treatments within the range of curability, growth delay can be directly assigned only to tumours which are seen to recur; *i.e.* to the sub-group least affected by the treatment. The sub-group most affected by the treatment ("cured" tumours) do not recur and therefore provide no observed data for calculation of growth delay. Herein lies the potential for bias by neglecting "cured" tumours in growth-delay experiments, and thus the need for some statistically fair way of taking account of them.

*The homogeneous Poisson model for tumour cure and recurrence*

Consider a population of identical tumours, each containing an identical number of clonogenic tumour cells, any one of which is capable of repopulating the tumour (note that this condition excludes immunogenic tumours). A true "cure" (no recurrence however long delayed) therefore requires elimination of all clonogenic cells, so that each cured tumour contains no surviving clonogenic cells after treatment.

However, for most cytocidal agents (radiation, most drugs) cell kill is a random process, whereby, at any given dose level, each clonogenic cell has a probability of being eliminated or of surviving. Hence, in a group of tumours given the same treatment, a proportion of tumours will be cured (no surviving clonogenic cells) and a proportion will recur (at least one surviving clonogenic cell).

Thus, in a group of $K$ treated tumours, the first tumour may have $N_1$ surviving cells, the second $N_2$ and so on, the $K$th tumour having $N_K$ cells, some of these $N$ values (those for the cured tumours) being zero.

Let $\bar{N}$ denote the arithmetic average for the whole group.

This is a situation to which Poisson statistics is readily applied, and it has been shown by several authors (*e.g.* Munro & Gilbert, 1961) that, if $\bar{N}$ is the average clonogenic cell number per tumour for a tumour group, then $P_c$, the probability of cure for an individual tumour (*i.e.* the probability that a particular tumour contains zero clonogenic cells when the group average is $\bar{N}$) is given by:

$$P_c = \exp.(-\bar{N}) \qquad (1)$$

The probabilities that a particular tumour contains larger numbers of cells may be found from higher terms in this Poisson expansion.

This implies that the average number of surviving clonogenic cells per tumour may be computed from knowledge of the probability of cure, $P_c$ (in practice, the proportion cured), *viz.*

$$\bar{N} = -\ln P_c \qquad (2)$$

The first two columns of the Table show the relationship of $\bar{N}$ to $P_c$ for a range of $P_c$ values from 0·05 (5% cured) to 0·99 (99% cured). It should be noted that $\bar{N}$ is exactly 1 when $P_c$ is 0·37 (*i.e.* at the $TCD_{37}$ dose level) and that $\bar{N}$ is fractional for all higher values of $P_c$. Of course, a fractional value of $\bar{N}$ does not imply that any particular tumour has a fraction of a clonogenic cell; fractional values arise by averaging over all tumours, some of which have (say) 1 or 2 cells, whilst other (cured) tumours have no clonogenic cells left. On this basis, there is no limit to how small $\bar{N}$ can become; in a group of 100 tumours, for example, of which 99 were cured (no cells) and 1 recurred from a single cell, the value of $\bar{N}$ would be 0·01.

By contrast, those tumours which actually do recur must have had at least one surviving clonogenic cell. If we denote by $\bar{N}_R$ the mean number of clonogenic surviving cells per tumour averaged over the sub-group of recurrent tumours only, it is clear that $\bar{N}_R$ could be never less than unity. Indeed, there is a simple relationship between $N$, $N_R$ and $P_c$:

$$\bar{N}_R = \frac{\bar{N}}{1 - P_c} \qquad (3)$$

This formula essentially modifies the group average $\bar{N}$ by excluding the proportion ($P_c$) of tumours for each of which the surviving cell number is 0.

The values of $\bar{N}$ and $\bar{N}_R$ corresponding to a range of values of $P_c$ are contrasted in the Table, the last column of which gives the ratio of $\bar{N}_R$ to $\bar{N}$, which reflects the magnitude of the difference.

TABLE.—*Mean number of surviving cells per tumour for all tumours* ($\bar{N}$) *and for recurrent tumours* ($\bar{N}_R$) *as a function of proportion cured* ($P_c$) *in homogeneous system* (*tumours and dosages*).

| $P_c$ | $\bar{N}$ | $\bar{N}_R$ | $\bar{N}_R/\bar{N}$ |
|---|---|---|---|
| 0·05 | 3·00 | 3·16 | 1·05 |
| 0·10 | 2·30 | 2·56 | 1·11 |
| 0·20 | 1·61 | 2·01 | 1·25 |
| 0·30 | 1·20 | 1·71 | 1·43 |
| 0·37 | 1·00 | 1·59 | 1·59 |
| 0·50 | 0·69 | 1·38 | 2·00 |
| 0·75 | 0·29 | 1·16 | 4·00 |
| 0·95 | 0·05 | 1·00 | 20 |
| 0·99 | 0·01 | 1·00 | 100 |

FIG. 1.—Relation of the ratio of the mean number of cells per recurrent tumour ($\bar{N}_R$) to the mean number of cells per tumour ($\bar{N}$), as a function of probability of cure ($P_c$), for a perfectly homogeneous tumour system. The points shown are the individual data points obtained by simulation.

As may be seen from the Table, and graphically, from Fig. 1, the magnitude of the ratio $\bar{N}_R/\bar{N}$ increased rapidly beyond a $P_c$ of about 0·5, becoming very large at high levels of tumour cure. Indeed, the factor has no finite limit and is theoretically infinite at total (100%) curability.

It is this factor which causes the bias when cured tumours are ignored in the results of growth-delay experiments.

*Effect of inter-tumour heterogeneity on distributions of surviving clonogenic cells*

In the preceding analysis, it was explicitly assumed that the tumour system was perfectly homogeneous (*i.e.* all tumours identical, all treatments identical). This is, of course, an unattainable ideal to which real systems approximate more or less well. More generally, inter-tumour heterogeneity will occur, and it is important to consider its effects. Inter-tumour heterogeneity, in the form of variable sensitivity to treatment, or, equivalently, of treatment dose received, is especially likely to occur in the case of treatment modalities for which uniformity of dose is difficult to achieve (*e.g.* hyperthermia, cytotoxic chemotherapy) resulting in the non-equivalence of individual tumours within the same treatment group.

In the present study, the effect of inter-tumour heterogeneity has been investigated using a Monte Carlo simulation model, described in the Appendix. Briefly, each individual tumour was assigned a sensitivity parameter ($D_0$) randomly selected from a normal distribution of $D_0$ values with specified mean and standard deviation. The standard deviation of the sensitivity distribution then provides a measure of the level of heterogeneity. The theoretical fraction of surviving cells for each individual tumour subjected to a given treatment dose was calculated using a multi-target function for the randomly assigned $D_0$; the theoretical surviving cell number (which could be an integer or fractional) was then taken as the mean of a Poisson distribution, and a random number selected from this distribution to represent the actual number of surviving cells (an integer or zero) for each tumour. Tumours assigned zero surviving cells were deemed cured and those assigned at least one surviving cell were deemed recurrent.

By carrying out a large number of such simulations for a range of treatment doses, and by varying the standard deviation of the sensitivity ($D_0$) distribution, it was possible to study the effects of the simulated heterogeneity (*i.e.* standard deviation of the $D_0$ distribution) on the numbers of surviving cells per tumour ($\bar{N}$) and per current tumour ($\bar{N}_R$) as a function of the proportion ($P_c$*) of cures obtained in each treatment group.

Fig. 2.—The effect of heterogeneity on the ratio of $\bar{N}_R$ to $\bar{N}$. Heterogeneity is generated by random selection of a $D_0$-value for each tumour from a normal distribution of $D_0$. The fractional standard deviation of the distribution of $D_0$ (s.d./mean %) provides a measure of the heterogeneity. The graph shows smooth curves drawn through large numbers of data points (not shown) obtained by simulation. The symbols are for indentification only.

The results, depicted in Fig. 2, show that inter-tumour heterogeneity may have a profound effect on the numbers of surviving cells, averaged over all tumours, or over recurrent tumours only. The discrepancy factor $\bar{N}/\bar{N}_R$ is strongly influenced by the heterogeneity and increases with the fractional standard deviation of the sensitivity distribution. It is not difficult to see how this comes about. In heterogeneous systems, the more sensitive tumours are cured at treatment dose levels which leave relatively large numbers of surviving cells in the more resistant tumours. Since the recurrent tumours are likely to be the more resistant ones, a large discrepancy may develop between the average number of surviving cells per tumour ($\bar{N}$) and per recurrent tumour ($\bar{N}_R$). The practical effect of this is that the presence of tumour cures distorts growth-delay experiments, which become increasingly distorted as heterogeneity increases.

*Effects of the discrepancy factor* ($\bar{N}/\bar{N}_R$) *on growth delay*

In order to proceed from numbers of surviving cells to growth delay, it is first necessary to determine the growth law for the tumour. For experimental tumours, the available evidence indicates a composite growth curve with an exponential (latent) phase giving way to a decelerating Gompertzian phase (see Steel, 1977 for discussion). The Gompertzian phase may, in itself, lead to some complications in the interpretation of growth delay experiments (Begg, 1980).

However, for most experimental tumours, growth is exponential for most of the growth range (*e.g.* from 1 cell to $10^6$ or $10^7$ cells). The present analysis is restricted to the case of purely exponential regrowth. It is unlikely that this restriction seriously affects the conclusions reached.

With the assumption of exponential regrowth it is possible to determine the tumour regrowth delay corresponding to particular numbers of surviving cells. Thus, for exponential growth at specific rate $\lambda$, a tumour-cell population which has been reduced to $N$ surviving clonogenic cells by the treatment will regrow to a population size of $N_0$ cells in $\tau_R$ where:

$$N_0 = N \exp(\lambda \tau_R) \qquad (4)$$

Let us now consider individual tumours to be coded 1, 2, 3, . . .*i*. . .*m* where there are *m* recurrent tumours in the group. Then $\tau_R^{(i)}$, the regrowth delay for the *i*th tumour is given by:

$$\tau_R^{(i)} = \frac{1}{\lambda} \ln (N_0/Ni) \qquad (5)$$

where $Ni$ is the number of surviving clonogenic cells for the *i*th tumour. The mean regrowth delay, $\bar{\tau}_R$ for the recurrent tumours is given by the arithmetic average of the regrowth delay times $\tau_R^{(i)}$, $\tau_R^{(2)} \ldots \tau_R^{(i)} \ldots \tau_R^{(\;)}$ for the *m* individual recurrent tumours. (Note that because of the logarithmic relationship between growth delay and cell number, the arithmetic average growth delay corresponds to a geometric average of cell number.)

Equation (5), used with the surviving cell numbers previously generated by the Monte Carlo procedure, allows calculation of the mean growth delay for the recurrent tumours at each dose. Since, in reality, only tumours which are recurrent yield measurable growth delay, this growth delay ($\bar{\tau}_R$) is the growth delay which would be observed experimentally.

Suppose, however, that a tumour was capable of regrowing from *less* than 1 cell (*e.g.* 0·1 cell or 0·01 cell). Such a tumour would, of course, have a longer growth delay than the longest physically possible time (for growth from a single cell) and this could be calculated using a similar equation to equation (7). Fractional mean levels of cell survival do indeed result from calculation of the effects of large treatment doses, using any continuous survival model. (*E.g.*, an initial population of $10^8$ cells would be reduced to 0·1 cells by a treatment giving a $10^{-9}$ surviving fraction.)

FIG. 3.—The relation of the "cure-correction factor" ($F$) (see text) to proportion cured ($P_c$*) for various levels of heterogeneity. Heterogeneity is indicated by the % s.d. of the $D_0$ distribution. The graph shows smooth curves drawn through large numbers of data points (not shown) obtained by simulation. The symbols are for identification only.

Though physical regrowth from less than one cell is, of course, biologically impossible, the theoretical regrowth delay time is easily calculated, so that the "regrowth time" of cured tumours can be included in the analysis. When the regrowth delay of all tumours (including those deemed cured) are calculated in this way, the individual growth delay may then be averaged as before to yield $\bar{\tau}$, the mean growth delay for all tumours in the group.

In this way it is possible to calculate a theoretical mean growth delay for all tumours and to compare it with the corresponding growth delay for the recurrent tumours only. To facilitate comparison, it is useful to define a "cure correction factor" ($F$) given by:

$$F = \bar{\tau}/\bar{\tau}_{R} \quad (6)$$

or

$$F = F/\bar{\tau}_{R} \quad (7)$$

*i.e.* the correction factor $F$ is the quantity by which the observed mean regrowth delay ($\bar{\tau}_R$) must be multiplied, in order to obtain the theoretical mean regrowth delay ($\bar{\tau}$) which takes account of cured tumour.

Using the mathematical model and Monte Carlo simulation procedure described in the Appendix, the cure-correction $F$ factor has been calculated as a function of the proportion of tumours cured ($P_c$*) in each dose group, and as a function of the heterogeneity of the system, *i.e.* as a function of the standard deviation of the sensitivity ($D_0$) distribution.

The results are shown in Fig. 3. As may be seen, the $F$ factor rises as a function of the cured proportion, and is also greater for greater heterogeneity. For perfectly homogeneous systems (zero variation of $D_0$), the factor rises slowly from 1·00 (at a zero proportion of tumour cures) to a value of 1·04 at a cured proportion of 0·50. This is a very modest correction, and implies that neglect of cured tumours produces no serious error in perfectly homogeneous tumour systems.

However, the magnitude of the correction factor increases with the heterogeneity. For a heterogeneity corresponding to 25% standard deviation of the sensitivity distribution, the $F$ factor has risen to 1·17 for a cured proportion of 0·50 and to 1·32 by a cured proportion of 0·80. This is a rather more serious correction and indicates that neglect of cured tumours in such a tumour system could lead to significant under-estimation of the effect of treatment.

It is instructive to compare these numerical properties of the $F$ factor with the "long delay" correction procedure suggested previously (Denekamp, 1980; Fowler *et al.*, 1980) to allow for cured tumours in growth-delay experiments. In this case, an "$F$ factor" for the "long delay" procedure (FLD) may be calculated by taking the ratio of the mean growth delay obtained by assigning a "long delay" (here taken to be the time to regrow from a single cell) to cured tumours, to the mean delay for recurrent tumours only.

For the heterogeneity levels previously considered, the "long delay" correction factor was compared with the factor derived here. For homogeneous systems, and for only slightly heterogeneous systems, these factors were both small in magnitude and did not differ significantly. As the heterogeneity increased however, the $F$ factor derived here increased rapidly

FIG. 4.—A comparison of the cure-correction factor ($F$, ●) with the corresponding factor (FLD, ○) obtained using the "long delay" correction procedure, for a $D_0$ distribution with a s.d. of 25%. The points shown are the individual data points obtained by simulation.

and more consistently than the "long delay" factor. This effect is illustrated in Fig. 4, which compares the two correction factors for heterogeneity corresponding to 25% standard deviation in the sensitivity ($D_0$) distribution.

Evidently, the "long delay" procedure could seriously underestimate the importance of cured tumours in growth-delay experiments involving the more heterogeneous tumour systems.

*A possible approach to the quantitative assessment of heterogeneity*

There seems little prospect of precisely correcting for cured tumours in growth-delay experiments unless the heterogeneity present can be quantified. This seems a daunting prospect, but an approach to the problem may be possible.

In a perfectly homogeneous system, no cures should be observed until the mean number of surviving cells per tumour becomes very low. Thereafter, a small increase in treatment dose should lead to a rapid rise in the proportion of cures. In heterogeneous systems, however, the more sensitive tumours will be cured whilst the more resistant tumours regrow from appreciable numbers of surviving

FIG. 5.—The effect of heterogeneity on the relation of observed mean regrowth delay ($\bar{\tau}_R$) to proportion cured. Heterogeneity is indicated by the % s.d. of the $D_0$ distribution. The graph shows smooth curves drawn through large numbers of data points (not shown) obtained by simulation. The symbols are for identification only.

cells. Considerable increase in treatment dose may be necessary before the more resistant tumours are cured.

These considerations suggest that comparison of the observed regrowth delay (for recurrent tumours) to the proportion cured may indicate the heterogeneity present in the tumour system. This is illustrated in Fig. 5, which shows the relation of observed growth delay to proportion cured for varying levels of heterogeneity. As expected, the observed growth delay rises steeply to its plateau value (the time to regrow from a single cell) for homogeneous systems, but the approach to the plateau level becomes progressively less steep as the heterogeneity increases. It is possible, therefore, that the shape of the curve relating observed growth delay to proportion cured could be used to indicate the level of heteterogeneity.

At the very least, a steep rise to the plateau level of growth delay provides confidence that the system under study is relatively homogeneous (and that neglect of cured tumours in growth-delay experiments is relatively unimportant) whilst a shallow rise provides a warning of possible heterogeneity (and a potentially serious effect of neglecting tumour cures). Since, in general, homogeneous experimental systems are preferable to heterogeneous ones, this may provide a further criterion for judging the suitability of the current experimental system.

However, further work is necessary to determine the practical feasibility of this approach, and the extent to which complicating factors may intrude. Amongst the complicating factors which require to be considered are the growth-kinetic changes which may accompany treatment (McNally, 1974; Brown & Howes, 1974; Stephens & Peacock, 1977) and which are already known to complicate the interpretation of growth-delay experiments. The effects of growth-rate variations amongst regrowing tumours, and the importance of sample size also require further consideration.

However, it seems possible that the relation of growth delay to cure could provide a rough estimate of heterogeneity, and such an approach may be worth further investigation.

### CONCLUSIONS

(1) In homogeneous tumour systems, the observed regrowth delay rises steeply, with proportion cured, to a plateau level. In such systems, only a small correction need be applied to the observed growth delay to allow for cured tumours; this correction is equally well made using the "long delay" procedure or from information presented in this paper.

(2) In non-homogeneous systems, the relation of observed delay to proportion cured rises more slowly. In such systems, neglect of cured tumours in growth-delay experiments may lead to a serious underestimation of the effectiveness of treatment. The "long delay" procedure will, in general, provide correction factors which are too small, again leading to underestimation of the effectiveness of treatment. Accurate correction factors cannot be derived without knowledge of the heterogeneity of the system.

(3) It is possible that the relationship of observed growth delay to proportion cured might provide estimates of the level of heterogeneity. Then, accurate correction factors could be derived. It is also possible that such information could be used to allow the reliable estimation of clonogenic cell survival *in situ*.

Acknowledgement is due to the participants of the 9th L. H. Gray Conference (Cambridge, September 1979) who took part in discussions at which the problem considered here was posed. We are especially grateful to Professor J. F. Fowler (C. R. C. Gray Lab, Northwood) for helpful suggestions and to Professor Fowler and Dr A. S. Michalowski (M.R.C. Cyclotron Unit, Hammersmith Hospital) for critical reading of the manuscript.

### REFERENCES

BEGG, A. C. (1980) Analysis of growth delay data: Potential pitfalls. *Br. J. Cancer*, **41**, Suppl. IV, 93.

BROWN, J. M. & HOWES, A. E. (1974) Comparison of tumour growth delay with cell survival. *Br. J. Radiol.*, **47**, 509.

DENEKAMP, J. (1980) Is any single *in situ* assay of tumour adequate? *Br. J. Cancer*, **41**, Suppl. IV, 56.

FOWLER, J. F., HILL, S. A. & SHELDON, P. W. (1980) Comparison of tumour cure (local control) with regrowth delay in mice. *Br. J. Cancer*, **41**, Suppl. IV, 102.

KALLMAN, R. F. & BEGG, A. C. (1980) Chairman's summary of Section II: Experimental studies *in situ*. *Br. J. Cancer*, **41**, Suppl. IV, 109.

McNALLY, N. J. (1974) Tumour growth delay and cell survival *in situ*. *Br. J. Radiol.*, **47**, 510.

McNALLY, N. J., DENEKAMP, J., SHELDON, P. W. & FLOCKHART, I. R. (1978) Hypoxic cell sensitization by misonidazole *in vivo* and *in vitro*. *Br. J. Radiol.*, **51**, 317.

MUNRO, T. R. & GILBERT, C. W. (1961) The relation between tumour lethal dose and the radiosensitivity and clonogenic cell number. *Br. J. Radiol.*, **50**, 843.

STEEL, G. G. (1977) *Growth Kinetics of Tumours*. Oxford: Clarendon Press.

STEPHENS, T. C. & PEACOCK, J. H. (1977) Tumour volume response, initial cell kill and cellular repopulation in B16 melanoma treated with cyclophosphamide and CCNU. *Br. J. Cancer*, **36**, 313.

### APPENDIX

Monte Carlo methods comprise that branch of experimental mathematics which may be used to simulate random physical processes using sequences of suitably distributed random numbers, and takes place using a computer rather than a laboratory.

The present investigation uses random numbers, first to introduce known degrees of heterogeneity in radiosensitivity into otherwise identical populations of tumours, and then to simulate the response of the individual members of these "experimental" populations to the random physical process of radiation cell kill, modelled by Poisson statistics.

### *Random number generator*

The random numbers used depend upon a common source of uniformly distributed random numbers, generated using a multiplicative congruential generator (modulus $2^{47}$ and generator $5^{15}$), investigated by Coveyou & MacPherson (1967); and upon a number of machine-independent computer subroutines described and tested by McGrath & Irving (1975), here implemented on a Data General Nova 1200 Computer in Fortran.

### *Tumour system*

The tumour system studied in these simulations is characterized by the following

"typical" parameter values:

Radiosensitivity $(D_0) = 3{\cdot}0$ Gy.
Extrapolation number $(n) = 2$.
Fraction of cure dominating clonogens $(\beta) = 0{\cdot}05$.
Initial tumour-cell population (before irradiation) against which the regrowth delay is assessed $(N_0) = 10^9$ cells.
Exponential tumour-doubling time $(T_D) =$ 2 days.

*Simulations*

From this set of parameters 9 distinct "experimental" populations were constructed, each comprising 50 tumours, whose individual radiosensitivities $(D_{0i})$ are randomly selected from a normal distribution of $D_0$ values with a mean value of 3·0 Gy and, for each population, a certain heterogeneity, $H$, expressed as s.d. as a percentage of this mean. Computationally, the random selection of elements from a normal distribution with a known mean $(D_0)$ and standard deviation $(HD_0)$ was made with an efficient algorithm written by Marsaglia & Bray (1964). The responses of *each* of these 9 populations to a range of single radiation doses was simulated in a process which, if carried out in the laboratory, would require at least 204 separate irradiations and the use of at least 10,200 tumour bearing animals.

For each individual tumour characterized by its randomly attributed radiosensitivity, $D_{0i}$, the average number of cells, $\bar{N}_i$, expected to survive a single radiation dose, $D$ (*were there many such identical tumours*); may be calculated theoretically from the high-dose approximation to the multitarget equation:

$$\bar{N}_i = \beta n N_0 \exp. (-D/D_{0i}) \qquad (8)$$

Allowance for the random nature of radiation cell killing and the fact that each tumour is considered individually, is made by using this theoretical average surviving cell number, $\bar{N}_i$, to define a Poisson distribution for *each* tumour from which the *actual* number of surviving cells, $N_i$, in that tumour, is randomly selected.

Computationally, the random selection of elements from a Poisson distribution was effected using a subroutine described by McGrath & Irving (1975) except in those cases where $\bar{N}_i > 10$ cells, where an approximating normal distribution was used in the interests of efficiency.

For each population and dose level, those tumours assigned zero cells by this process, were considered cured and the corresponding proportion termed "the observed cure probability", $P_c{*}$. The radiation dose range used for each population was adjusted so that this probability always encompassed the range 0·1–0·9. Dose increments of 1·0 Gy were adhered to throughout.

For each population and dose level, those tumours assigned one or more surviving cells were assumed to recur, constituting a population subgroup from which "observations" of regrowth delay are made. The geometric *average* cell number $\bar{N}_R$, in this subgroup may be obtained from the relation:

$$\ln \bar{N}_R = \frac{1}{M} \sum_{i=1}^{M} \ln N_i; \qquad N_i \neq 0 \qquad (9)$$

where $M$ is the number of recurrent tumours in a given population. The dependence of the discrepancy factor $(\bar{N}_R/\bar{N})$ upon the degree of population heterogeneity may now be investigated (see Fig. 2).

### REFERENCES

Coveyou, R. R. & MacPherson, R. D. (1967) Fourier analysis of uniform random number generators. *J. ACM*, **14**, 100.

Marsaglia, G. & Bray, T. A. (1964) A convenient method for generating normal variables. *SIAM Rev.*, **6**, p. 1.

McGrath, E. J. & Irving, D. C. (1975) *Techniques for efficient Monte Carlo Simulation. Vol. 2. Random number generation for selected probability distributions.* Tennessee: Oak Ridge National Laboratory.